# Burn Ointment Promotes Cutaneous Wound Healing by Modulating the PI3K/AKT/mTOR Signaling Pathway

**DOI:** 10.3389/fphar.2021.631102

**Published:** 2021-03-08

**Authors:** Dali Gan, Qiyuan Su, Hanwen Su, Li Wu, Jun Chen, Bing Han, Meixian Xiang

**Affiliations:** ^1^ School of Pharmaceutical Sciences, South-Central University for Nationalities, Wuhan, China; ^2^ Department of Statistics, University of Illinois at Urbana-Champaign, Urbana, IL, United States; ^3^ Department of Clinical Laboratory, Renmin Hospital of Wuhan University, Wuhan, China; ^4^ Department of Pharmacy, Wuhan No.1 Hospital (Wuhan Hospital of Traditional Chinese and Western Medicine), Wuhan, China; ^5^ Department of Pathology, Penn State College of Medicine, Milton S. Hershey Medical Center, Hershey, PA, United States

**Keywords:** burn ointment, safety evaluation, burn wounds healing, analgesia, anti-inflammatory, PI3K/AKT/mTOR pathway

## Abstract

Burn ointment (BO) is a clinically useful medicine for the treatment of burns and scalds. However, there is no enough scientific evidence to report the effect of BO on wound healing and its analgesic and anti-inflammatory efficacy. The aim of this work was to evaluate the anti-inflammatory and analgesic efficacy of BO and to reveal the potential wound healing properties and related mechanisms of BO. In this work, the content of active ingredients of BO was determined by high-performance liquid chromatography (HPLC). Two animal models of inflammation were used to study its anti-inflammatory activity, and a hot plate method was used to evaluate its analgesic effect. In addition, mouse incision and rat burn models were used to investigate the effect of BO on the anti-inflammatory and wound healing mechanisms. The results showed that BO was safe for topical application, and BO could significantly inhibit auricular swelling in mice and paw swelling in rats and significantly prolong the latency period of paw licking in the hot plate experiment in mice. It can also accelerate wound healing and repair scars by promoting the formation of new epithelial tissues in rat burn models. In addition, BO significantly downregulated the serum level of TNF-α and significantly increased the serum levels of VEGF and TGF-β1. Also, BO promoted the expression of collagen I and increased the ratio in p-PI3K/PI3K, p-AKT/AKT, and p-mTOR/mTOR pathways. Our results demonstrate the safety and efficacy of BO and suggest that activation of the PI3K/AKT/mTOR signaling pathway may play an important role in the promotion of wound healing by BO.

## 1 Introduction

Burns are one of the most serious skin-related injuries, and their incidence is on the rise globally (Shan et al., 2015). In the United States, the number of burn victims is estimated as 1.2 million per year. Of these injuries, an average of 50,000 burn victims are severely burned and require hospital treatment (Kaddoura et al., 2017).

**GRAPHICAL ABSTRACT:**
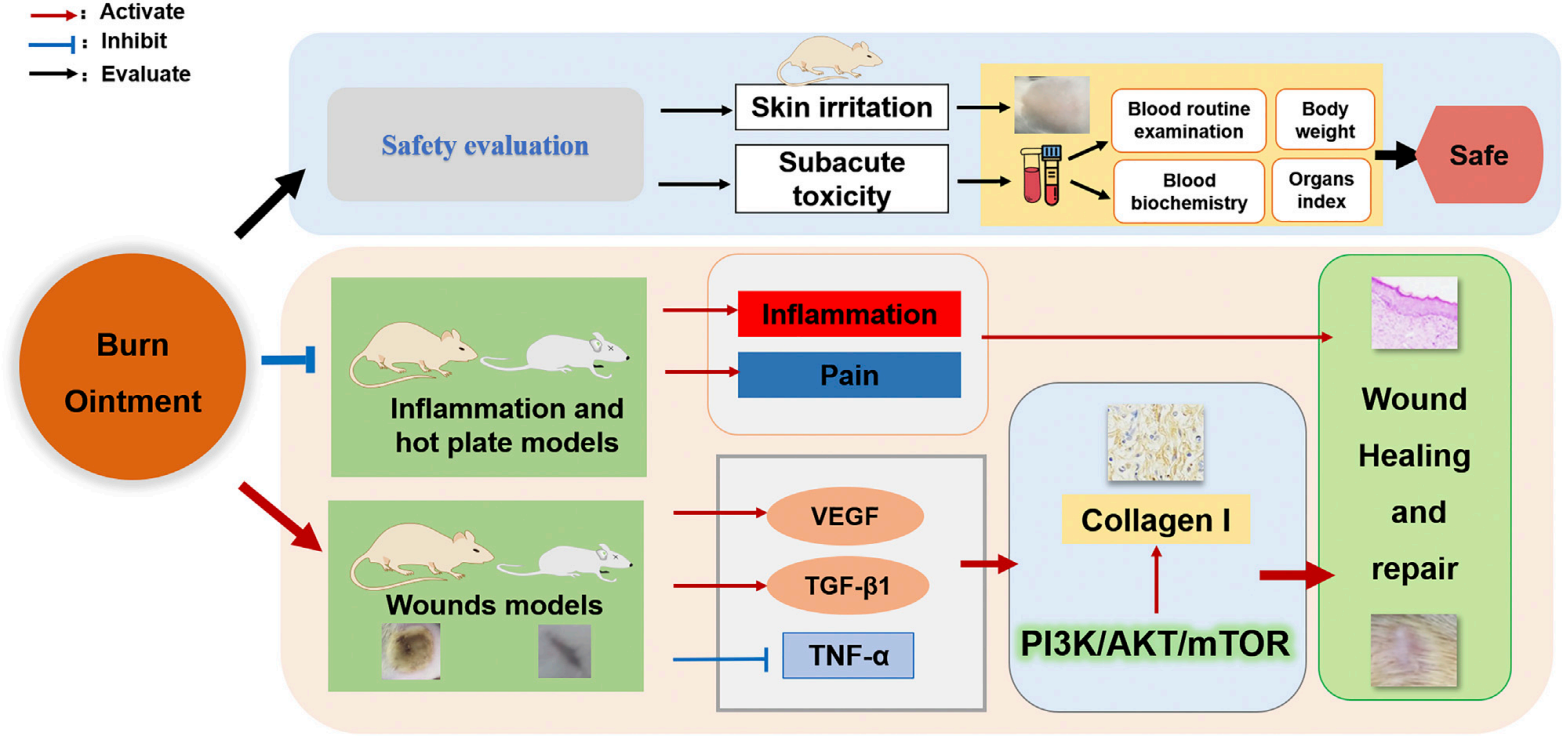


The largest organ of the human body is the skin, and it is able to resist external infections and maintain the stability of the internal environment of our body (Riepl, 2020). Mild scalding will damage the skin tissue, but severe burns will be life-threatening. Additionally, the burns can cause inflammation, pain, and other complications (Wang et al., 2018). There are three phases that can be concluded in the dynamic healing process of wounds, including the inflammatory phase, the proliferation phase, and the maturation phase.

Many researches have indicated that several key cellular factors are involved in the process of burn repair, such as tumor necrosis factor-alpha (TNF-α), vascular endothelial growth factor (VEGF), and transforming growth factor-beta1 (TGF-β1) (Kim et al., 2020; Yaseen et al., 2020). If the body acquires an infection when the skin is damaged, it is stimulated to secrete the proinflammatory cytokines, such as TNF-α. After a while, the cytokine initiates an inflammatory cascade reaction to remove necrotic tissues and cells (Zelova and Hosek, 2013). However, overactive inflammation can cause systemic inflammatory response syndrome and immune dysfunction in patients, which will endanger their lives (Boldeanu et al., 2020). Therefore, anti-inflammatory treatments are essential for burn wound healing processes. Once the inflammation subsides, macrophages and other cells begin to secrete growth factors, such as VEGF. VEGF is an essential cell factor for forming new blood vessels, and it can promote vascular endothelial cell proliferation to produce new blood vessels (Roskoski, 2017). The new blood vessels can provide nutrition to the injured area and accelerate wound healing (Eming et al., 2014). Besides, TGF-β1 is also a necessary cell factor for the healing process, because it promotes fibroblasts to proliferate and differentiate, forms granulation tissues, and synthesizes type Ι collagen (Kim et al., 2018a). Collagens help with wound contraction and restore skin elasticity to heal the burn wound by filling the space between cells (Gonzalez et al., 2016).

Currently, most of the known medications for burns have the obvious limitation of leaving scars easily. Traditional Chinese medicine compound burn ointment offers a more advanced alternative that avoids such limitations. BO is a traditional Chinese medical formulation, which contains *Rheum palmatum* L., *Angelica sinensis* (Oliv.) Diels, *Codonopsis pilosula* (Franch.) Nannf., *Asarum sieboldii* Miq., *Borneolum syntheticum*, and *Calomelas*. Traditionally, *Rheum palmatum* L. can be used to cure burn wounds *in vitro* (Committee of the Pharmacopoeia of PR China, 2015) due to its antimicrobial and anti-inflammatory qualities (Cao et al., 2017), and it can promote recovery of muscle; *Angelica sinensis* (Oliv.) Diels and *Codonopsis pilosula* (Franch.) Nannf. can remove stasis and analgesia (Wei et al., 2016; Gao et al., 2018), promoting the skin recovery of the wounded area. Modern research has revealed that the extract of *Rheum palmatum* L. could accelerate the progress of wound healing in rats, and its efficacy was better when it combined with the extract of *Angelica dahurica* (Fisch. Ex Hoffm.) Benth. et Hook. f (Yang et al., 2017). Moreover, many studies have shown the enormous potential of BO in treating burns as well, such that many chemicals of *Rheum palmatum* L. and *Angelica sinensis* (Oliv.) Diels could reduce the liberation of proinflammatory cytokines, including IL-1β, IL-6, TNF-alpha, and IL-33 (Kim et al., 2018b; Shen et al., 2019). They have an anti-inflammatory and analgesic effect (Wei et al., 2016), induce cell proliferation (Gundogdu et al., 2019), and protect cells from injuries (Zhang et al., 2019b). But, the activity of BO in anti-inflammation, analgesia, and promoting skin wound healing, and its specific mechanism are still unclear. Therefore, this study attempted to elucidate the efficacy, the involved molecular mechanisms of BO in the healing process of burn wounds, and their complications. This research would contribute to the development and application of new drugs to treat burns and scalds.

## 2 Material and Methods

### 2.1 Materials and Reagents

BO (pharmaceutical batch number: 201671HZ) was supplied by the Department of Pharmacy at the Wuhan Integrated Traditional and Western Medicine Hospital (Wuhan, China). The BO used in this study is a hospital preparation which contains *Rheum palmatum* L., *Angelica sinensis* (Oliv.) Diels, *Codonopsis pilosula* (Franch.) Nannf., *Asarum sieboldii* Miq., *Borneolum syntheticum,* and Calomelas. Burn moisturizing scald ointment (BMS) was purchased from Meibao Pharmaceutical Co., Ltd. (Shantou, China); ELISA kits were obtained from Neobioscience Technology Co., Ltd. (Shenzhen, China); Collagen I primary antibody; phosphorylated PI3K, AKT, and mTOR; unphosphorylated PI3K, AKT, and mTOR polyclonal antibody; and HRP-labeled secondary antibody were purchased from Servicebio (Wuhan, China); Microscope (XSP-C204) was from Motic China group and microscope camera was purchased from Cognex (Massachusetts, United States); Toe volume measuring instrument (PV-200) was from TECHMEN Co., Ltd. (Chengdu, China); full-wavelength microplate reader 1,510 was obtained from Thermo Fisher Scientific (Massachusetts, United States); Ultimate 3000 series HPLC system consisting of the computer-controlled system with the CHROMELEONTM software and a SQL database, equipped with a WPS-3000 autosampler, was from Dionex (California, United States).

### 2.2 Animals

Kunming (KM) mice (20 ± 2 g), Sprague Dawley (SD) rats (200 ± 20 g), and Sprague Dawley (SD) rats (90 ± 10 g) were purchased from the Experimental Animal Center (certificate of experimental animals: SCXK 2015–0089 for rats and SCXK 2015–0018 for mice), Institute of Health and Epidemic Prevention (Wuhan, China), and housed in the standard specific pathogen-free (SPF) environment for three days. License number of the experimental unit was SYXK (Hubei province) 2016–0089, Experimental Animal Center of Central South University for Nationalities. All animals were allowed free access to food and water. The animal experiments were approved by the Institutional Animal Ethical Committee of Central South University for Nationalities and were performed under the guidelines of the committee; the permit number for the use of animals was no. 2020-scuec-029.

### 2.3 HPLC Analysis of BO’s Chemical Components and Stability

20 mg BO was extracted with 10 ml methanol for 20 min using the ultrasonic method. The obtained extract was separated and transferred to a 10 mL volumetric flask and the volume was adjusted to 10 ml with methanol to obtain a 1 mg/mL sample solution. HPLC was then used to analyze the sample solution to quantify the content of aloe-emodin, emodin, rhein, chrysophanol, ferulic acid, lobetyolin, asarinin, and borneol and evaluate their stability on the 30th day or beyond. The samples were subjected to HPLC analysis on a C_18_ column (Agilent 250 mm × 4.6 mm, 5 μm) by a single injection of 10 μl detected at 254 nm; mobile phase: gradient elution by methyl alcohol (A) and water (B). The gradient program was set as follows: 0–4 min, 5–21% A; 4–8 min, 21–26% A; 8–10 min, 26–32% A; 10–14 min, 32–36% A; 14–18 min, 36–48% A; 18–22 min, 48–55% A; 22–25 min, 55–65% A; 25–30 min, 65% A; column temperature: 25°C; flow rate: 0.8 ml/min.

### 2.4 Doses and Methods of BO in Animal Models

Mice were randomly divided into several groups: canola oil (Con) group, burn moisturizing scald ointment (BMS) group, and BO group, with 10 mice in each group. The BO dosages of 0.1 g, 0.2 g, and 0.4 g/cm^2^ were used to assess the subacute toxicity.

### 2.5 Skin Irritation Experiment in Rats

One day before the experiment, twenty SD rats were shaved for an area of approximately 4 × 2 cm^2^ on the dorsal side. These shaved rats were randomly divided into two groups of ten: the canola oil group (Con) and the BO-treated group (BO). Then 0.2 g/cm^2^ BO or Con was applied evenly on the shaved area of animals three times per day for one day. One hour after the last treatment, the treated skin was cleaned with cotton and water. After the application of BO, any signs, symptoms, and other skin changes were observed for 4 h (Ghosh et al., 2019).

### 2.6 Subacute Toxicity Assessment

Forty SD rats were shaved, weighed, and categorized into four groups of ten: 0.1 g, 0.2 g, and 0.4 g/cm^2^ with BO and 0.2 g/cm^2^ with Con. All animals received BO or Con 3 times per day for a total of 28 days. The body weight was recorded once per week. At the end of the experiment, all animals were fasted for 12 h and then anesthetized for blood and organ collection (Upadhyay et al., 2009). Hematological and biochemical analyses were run in a clinical laboratory, Wuhan University Renmin Hospital.

### 2.7 The Hot-Plate Test

The hot-plate test was used to examine the potential analgesic effect of BO. First, 40 KM mice were screened for their pain threshold within 5–30 s. In brief, the temperature of the plate surface was kept at 54 ± 1°C. The interval time that the mice licked its toe or jumped from the plate was detected three times. Twenty-seven mice were selected and randomly divided into the following groups: the Con group, the BO group, and the BMS group. All mice were applied 0.2 g/cm^2^ drug each time on the hind paws, 3 times per day for 7 days. The response latency of mice at the first hind paw lick or jump was recorded and analyzed 30 min after the last dose on day 1 and day 7, respectively (Ariyo et al., 2020). The following formula was used to calculate the pain threshold change:

Pain threshold change (s) = Pain threshold after treatment (s) − Pain threshold before treatment (s).

### 2.8 The Xylene-Induced Mouse Ear Swelling

Thirty KM mice were randomly divided into three groups of ten: the Con, the BO, and the BMS groups. The animals' ears were treated with 0.2 g/cm^2^ drug each time, 3 times per day for 5 days. On the sixth day, 100 μl of xylene was dripped on the right ear with a pipette. After the model was established successfully, the animals were immediately sacrificed, and both ears were removed. The samples were immediately weighed after being punched (Xavier-Santos et al., 2018). The following formula was used to calculate the degree of the ear swelling:

The degree of ear swelling: Δm (mg) = The right ear mass (mg) − The left ear mass (mg).

### 2.9 Carrageenan-Induced Toe Swelling

Thirty SD rats were randomly divided into three groups of ten, the Con group, the BO group, and the BMS group, with 0.2 g/cm^2^ drug topically applied evenly on the right hind paw 3 times/day for a total of 5 days. On the sixth day, each rat's right hind paw was injected with 0.1 ml carrageenan (1%). Rats’ right hind paw was marked by black marker at its ankle, then, the paw volume before and after injection at 1–5 h was measured by Toes Volume Measuring Instrument, with three measurements each time point (Zahra et al., 2020). The following formula was used to calculate the toe swelling:

The swelling volume (ΔV) (ml) = V_t_ (ml) − V_0_ (ml), where Vt is the right hind paw volume (ml) at test time and V_0_ is the right bind paw volume (ml) before carrageenan injection.

### 2.10 Incision Wound Model

Twenty mice were randomly divided into two groups of ten, the Con group and the BO group. After dorsal fur shaving and anesthesia, a 1.5-cm long, lateral incision near the tail's root was made on the back. Then, 0.2 g/cm^2^ of Con or BO was topically applied over the incision three times per day for 16 days (Getachew et al., 2019). The changes in the wound were observed and recorded.

### 2.11 Thermal Burn Wounds Model

The dorsal hairs of 36 SD rats were shaved 24 h before the induction of burn. After disinfection and anesthesia, the thermal burn injuries were induced on the dorsal of rats by a metal block (1.5 cm^2^) heated over the flame for 10 s. After half an hour, all animals were awakened and randomly evenly divided into the Con group, the BO group, and the BMS group, with 0.2 g/cm^2^ drug topically applied each time, 3 times per day for 28 days. The wounded skin tissue was collected for H&E staining and Western blot test at the 1st, 7th, 14th, and 28th day after the model was established. Blood samples were collected from the abdominal aorta for TNF-α, VEGF, and TGF-β1 measurements, following the commercial kit instructions (Neobioscience, Shenzhen, China).

The wound contraction was evaluated by tracing the wound, using transparent paper and permanent marker, on the 1st, 7th, 14th, and 28th postwounding day. The wound area was retraced on the paper, and the changes in area were calculated, which indicated the wound contraction (Ghosh et al., 2019). The percentage of wound closure was calculated using this formula:

Wound contraction = [(wound area day1– wound area day n)/wound area day1] ×100%, n = 7th, 14th, and 28th after wound.

### 2.12 Histopathological Evaluation and Type I Collagen Staining on Burn Wound Tissue

The burn wound tissue was collected and H&E stained for histopathological evaluation. The pretreated sample tissues from rats were embedded in paraffin wax. The embedded wax blocks were then cut into 4-μm sections. The sections were routinely deparaffinized with xylene and different concentration alcohol. After the conventional staining, alcohol gradient dehydration, then, the samples were sealed with neutral resin. The observations included the epidermis, dermis, blood vessels, cells, and organelles. Two pathologists blindly read and evaluated histopathological changes with a scoring system. The detail score criteria included the regeneration of epithelial tissues, inflammatory cell infiltration, angiogenesis, and the thickness of the granulation; scores from 0 to 3 indicated from lack of recovery to full recovery. The four variables were summed to represent the histopathological score (total score: 0–12, respectively) (Kazemi et al., 2020).

The immunohistochemistry was applied to determine the type I collagen expression. In brief, after head-based antigen retrieval, each section was incubated with collagen I rabbit poly-Ab (Servicebio, 1:100), 4°C overnight. Then these sections were incubated with HRP-labeled goat anti-rabbit Ab (Servicebio, 1:200) for 2 h at room temperature. Finally, the section was stained with DAB and counterstained with hematoxylin. The sections were observed and photographed. The type I collagen expression was analyzed using ImageJ software (National Institutes of Health, Bethesda, MD, United States). The immunohistochemistry (IHC) Image Analysis Toolbox Plug-tool was first used to identify areas of positive staining. Thirty-six images were then converted to the RGB scale and the threshold tool was used to calculate the percentage area positively stained (SenthilKumar et al., 2020).

### 2.13 Western Blot Analysis

The protein samples (30 µg) from wound skin tissue lysates of burn rats were concentrated and separated respectively by 5 and 10% SDS-polyacrylamide gel electrophoresis (PAGE). Then, they were electrotransferred to PVDF membranes. Then, the membranes were blocked with a Western blocking solution (Tris-buffered saline with 0.05% Tween-20 and 5% nonfat dry milk) for 90 min and incubated at 4°C overnight with primary antibodies, phosphorylated and unphosphorylated PI3K, AKT, and mTOR polyclonal antibody (1:1000, Servicebio, Wuhan, China), followed by washing 3 times in TBST, 5 min/time and incubation with the HPR-labeled goat anti-rabbit secondary antibodies (1:1000, Servicebio, Wuhan, China) at room temperature for 1 h. The membranes were washed 3 times in TBST, 5 min/time. Finally, the protein levels were detected by gel imaging analyzing system (Bio-Rad, California, United States) and normalized to GAPDH.

### 2.14 Statistical Analysis

The data were expressed as the means ± standard error of mean (SEM). Statistical analysis was performed via Student's *T* test and one-way ANOVA (SPSS Program, version 11.5; SPSS, Inc., Chicago, IL, United States). Significant differences were considered when *p* < 0.05.

## 3 Results

### 3.1 Result of HPLC Analysis

**FIGURE 1:**
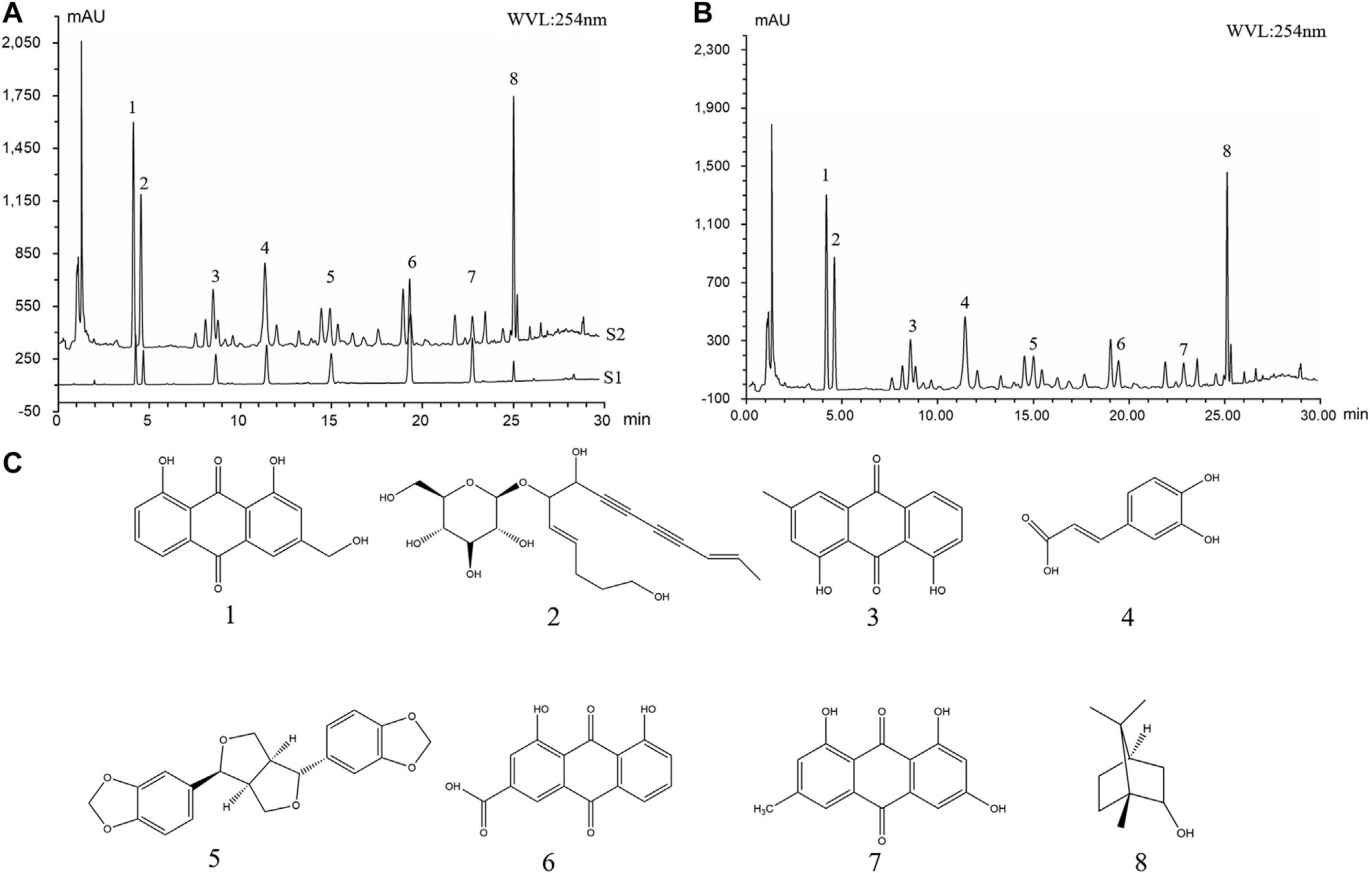
High-performance liquid chromatography (HPLC) analysis of the standard and the BO extract. **(A)** The content determination of standards (S1) and sample (S2). **(B)** The stability of BO after 30 days. **(C)** Compound structure. 1. Aloe-emodin; 2. lobetyolin; 3. rhein; 4. ferulic acid; 5. asarinin; 6. emodin; 7 chrysophanol; 8. borneol.

HPLC was used to detect the active ingredients in the BO and its stability within 30 days. Eight main peaks were obtained, indicating aloe emodin, emodin, rhein, chrysophanol, ferulic acid, lobetyolin, asarinin, and borneol, respectively. (Figure 1). The result was identical with the peaks of a single compound of aloe emodin, emodin, rhein, chrysophanol, ferulic acid, lobetyolin, asarinin, and borneol. And the result suggested that BO was stable within 30 days (Figure 1B).

### 3.2 Safety Assessment of BO

**FIGURE 2:**
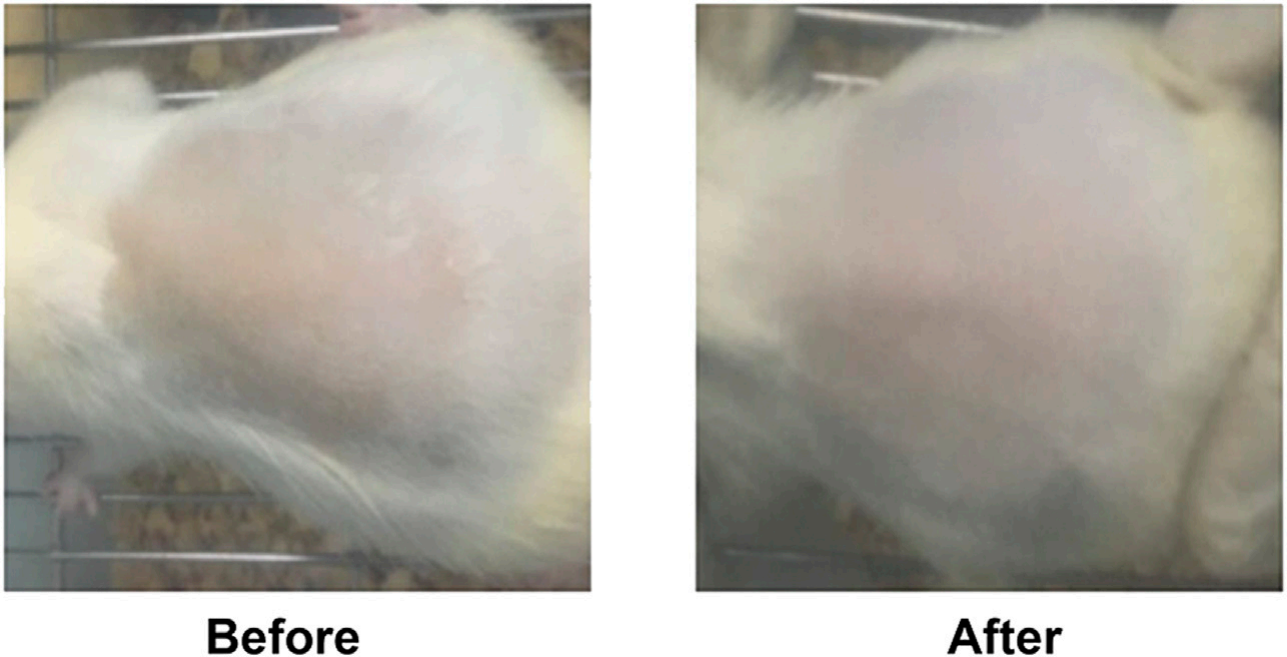
Representative imaging of rat skin before and 24 h after BO administration. 0.2 g/cm^2^ BO or Con was applied evenly on the shaved area of animals 8 h per time for 3 times. One hour after the last times, the skin condition was observed (n = 10 each group).

The skin irritation experiment in rats was used to evaluate the safety of BO. We did not observe significant differences in the exposed skin in terms of irritation, swelling, inflammation, redness, or other abnormal changes before and after treatment (Figure 2). Similarly, the preclinical trial for subacute toxicity showed no striking difference among rats treated with BO groups (0.1 g, 0.2 g, and 0.4 g/cm^2^) and Con group in rat body weight (Table 1), the relative organ weight (Table 2), blood routine (Table 3), and biochemical analysis (Table 4). Thus, our results indicated that BO was safe for rats.

**TABLE 1:** Changes of weight in rats (n = 10, mean ± SEM).

Days	Con	BO		
		0.1 g/cm^2^	0.2 g/cm^2^	0.4 g/cm^2^
1	100.75 ± 0.99	100.63 ± 1.65	102.63 ± 1.09	103.63 ± 1.83
7	127.25 ± 1.56	132.25 ± 2.44	131.13 ± 1.88	135.25 ± 3.50
14	196.50 ± 1.58	200.88 ± 4.10	207.88 ± 3.73	199.88 ± 5.35
21	239.38 ± 2.32	251.13 ± 6.24	261.13 ± 4.78	252.00 ± 4.52
28	288.25 ± 3.10	300.50 ± 8.42	310.38 ± 5.16	302.38 ± 6.00

Con: canola oil and BO: burn ointment. The data are expressed as the mean ± SEM (n = 10), compared with the Con group; *p < 0.05, **p < 0.01.

**TABLE 2:** Effect of BO on rat organ coefficient (mg/g) (n = 10, mean ± SEM).

Organs	Con	BO		
		0.1 g/cm^2^	0.2 g/cm^2^	0.4 g/cm^2^
Heart	3.38 ± 0.02	3.60 ± 0.09	3.65 ± 0.07	3.66 ± 0.08
Liver	27.22 ± 0.67	28.58 ± 2.29	28.12 ± 0.4	27.89 ± 0.42
Spleen	2.07 ± 0.02	1.94 ± 0.05	2.34 ± 0.09	2.15 ± 0.17
Lungs	4.19 ± 0.20	4.27 ± 0.15	3.93 ± 0.13	4.03 ± 0.01
Kidney L	3.45 ± 0.08	3.60 ± 0.04	3.49 ± 0.01	3.69 ± 0.09
Kidney R	3.51 ± 0.03	3.72 ± 0.08	3.59 ± 0.08	3.79 ± 0.09
Testes L	6.65 ± 0.17	6.54 ± 0.05	6.26 ± 0.18	6.49 ± 0.02
Testes R	6.67 ± 0.19	6.58 ± 0.03	6.86 ± 0.01	6.53 ± 0.08
Thymus	1.64 ± 0.01	1.85 ± 0.09	1.71 ± 0.13	1.73 ± 0.06

Con: canola oil and BO: burn ointment. The data are expressed as the mean ± SEM (n = 10), compared with the Con group; *p < 0.05, **p < 0.01.

**TABLE 3:** Effect of BO on blood routine (n = 10, mean ± SEM).

	Con	BO		
		0.1 g/cm^2^	0.2 g/cm^2^	0.4 g/cm^2^
WBC (×109/L)	9.61 ± 1.18	10.45 ± 1.44	9.33 ± 1.37	8.34 ± 0.21
RBC (×1,012/L)	8.37 ± 0.13	7.67 ± 0.27	8.02 ± 0.23	8.35 ± 0.07
HB(g/L)	149.00 ± 2.39	147.67 ± 1.74	155.33 ± 1.59	160.67 ± 1.20
HCT (L/L)	0.46 ± 0.01	0.46 ± 0.00	0.48 ± 0.00	0.50 ± 0.00
MCV (fL)	54.60 ± 0.30	59.93 ± 1.55	60.03 ± 1.24	59.50 ± 0.65
MCH (pg)	17.80 ± 0.14	19.40 ± 0.44	19.43 ± 0.36	19.23 ± 0.15
MCHC (g/L)	326.00 ± 0.84	323.33 ± 1.32	324.00 ± 0.55	323.33 ± 1.80
PLT (×109/L)	1098.67 ± 46.97	896.33 ± 51.98	1184.33 ± 18.58	1140.00 ± 24.26
PCT (%)	1098.67 ± 46.97	896.33 ± 51.98	1184.33 ± 18.58	1140.00 ± 24.26
MPV (fL)	7.73 ± 0.08	7.90 ± 0.09	7.87 ± 0.10	7.80 ± 0.06
PDW (fL)	8.30 ± 0.11	8.47 ± 0.15	8.53 ± 0.08	8.40 ± 0.08
P-LCR (%)	9.53 ± 0.43	10.13 ± 0.69	10.10 ± 0.57	9.70 ± 0.46

WBC: leukocyte; RBC: erythrocyte; HB: hemoglobin; HCT: hematocrit value; MCV: mean corpuscular volume; MCH: mean corpuscular hemoglobin; MCHC: mean corpuscular hemoglobin concentration; PLT: platelet; PCT: procalcitonin; MPV: mean platelet volume; PDW: platelet distribution width; P-LCR: platelet-larger cell ratio; Con: canola oil, BO: burn ointment. The data are expressed as the mean ± SEM (n = 10), compared with the Con group; *p < 0.05, **p < 0.01.

**TABLE 4:** The effect of BO on blood biochemistry in rats (n = 10, mean ± SEM).

	Con	BO		
		0.1 g/cm^2^	0.2 g/cm^2^	0.4 g/cm^2^
ALT (U/L)	44.33 ± 1.11	45.00 ± 1.38	48.00 ± 1.92	40.67 ± 2.46
AST (U/L)	167.67 ± 4.79	191.33 ± 9.87	156.67 ± 4.13	142.33 ± 9.34
Urea (mmol/L)	7.67 ± 0.15	7.57 ± 0.21	9.23 ± 0.17	8.33 ± 0.05
Cr (μmol/L)	25.33 ± 1.46	24.33 ± 0.36	24.00 ± 0.84	22.33 ± 0.48

ALT: alanine transaminase; AST: aspartate transaminase; Cr, creatinine; Con: canola oil, BO: burn ointment. The data are expressed as the mean ± SEM (n = 10), compared with the Con group; *p < 0.05, **p < 0.01.

### 3.3 Effect of BO on Analgesic

**FIGURE 3:**
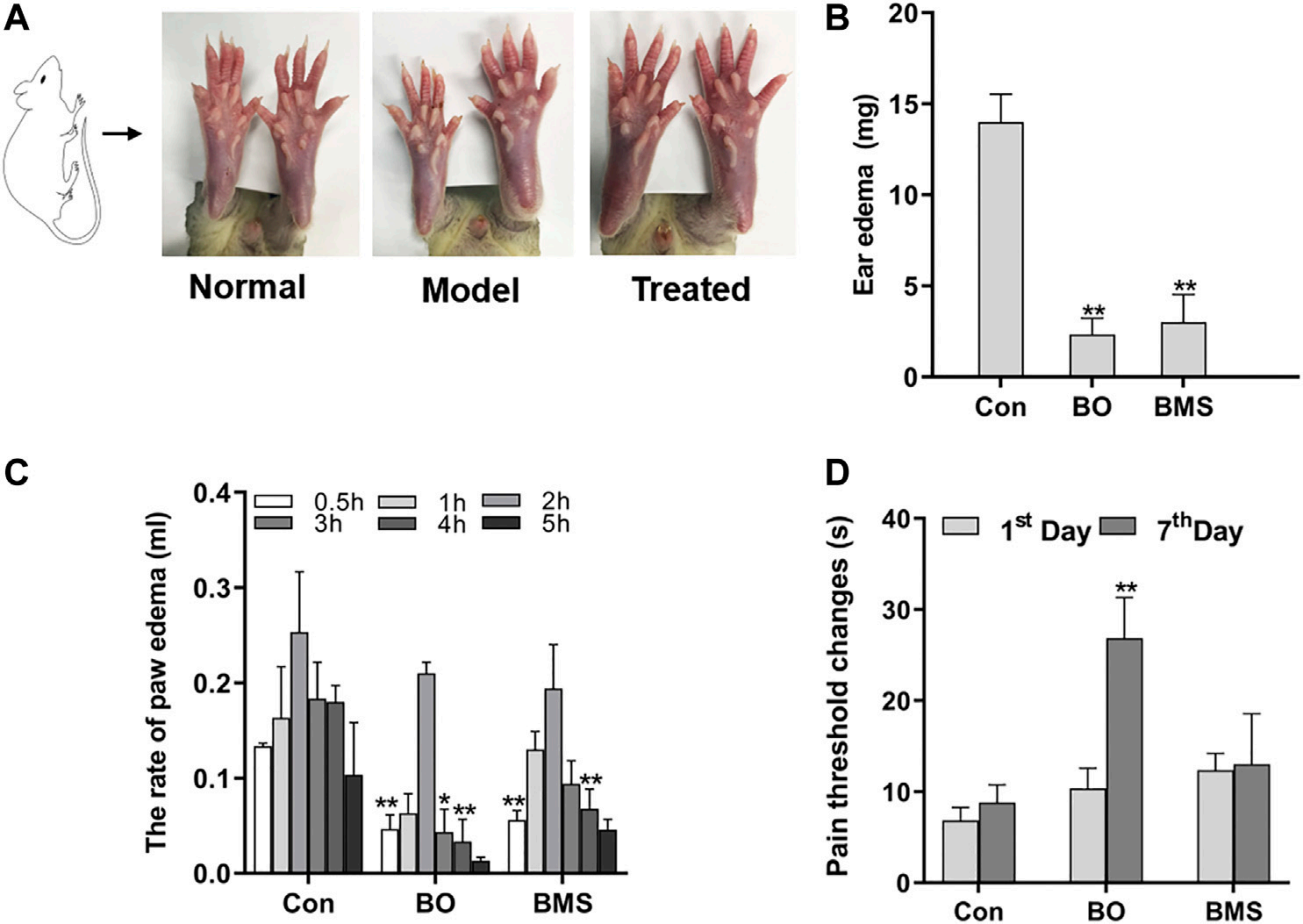
The anti-inflammatory and analgesic effects among Con, BO and BMS. **(A)** Representative imaging of rat toe before and after model induction, before and after treatment. **(B)** On the 6th day, BO pretreatment reduced mouse ear edema; the data were shown as the mean ± SEM (n = 10 each group), compared with the control group; *p < 0.05, **p < 0.01. **(C)** On the 6th day, BO pretreatment decreased rats’ toe swelling; the data are expressed as the mean ± SEM (n = 10 each group), compared with the control group; *p < 0.05, **p < 0.01. **(D)** BO pretreatment enhanced pain threshold; the data were expressed as the mean ± SEM (n = 9 each group), compared with the control group; *p < 0.05, **p < 0.01.

As revealed in Figure 3D, on the 7th day, the pain threshold of the BO group was significantly increased compared to that of the Con group (*p* < 0.01), while there was no difference in the Con and the BMS group. The results indicated that BO had an analgesic effect.

### 3.4 Effect of BO on Inhibiting Inflammation

In the model of carrageenan-induced toe swelling, we observed that the rats treated with BO demonstrated a significant reduction in swelling at 3 h after injection (*p < 0.05 or **p < 0.01, respectively) (Figure 3C). In the second model, auricle swelling was significantly reduced in the BO-treated group (**p < 0.01) (Figure 3B). These results indicated that BO could inhibit inflammation.

### 3.5 Effects of BO on Promoting Wounds Healing

**FIGURE 4:**
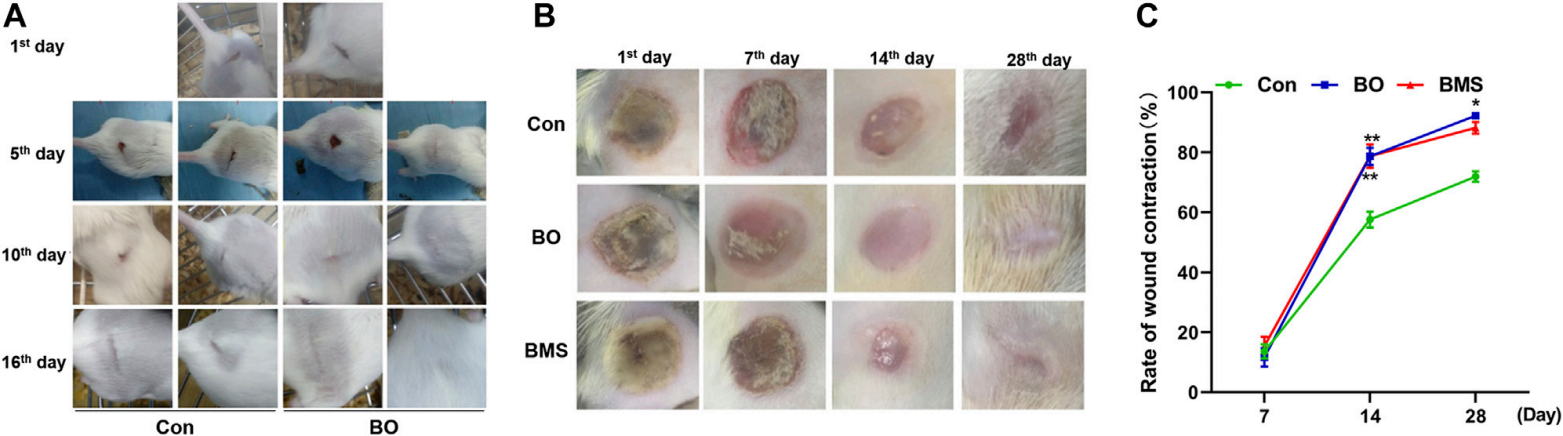
Representative imaging wound healing in mouse incision wound model and rat thermal burn wounds model, and the wound contraction quantification in the thermal burn wounds model. **(A)** Wound healing in mouse incision wound model. The healing process was observed and photographed on the 1st, 5th, 10th, and 16th day. The control group (Con) and the treatment group (BO), which were observed 3 times per day for 16 days (n = 10 each group). Representative pictures revealing that BO pretreatment accelerate the healing process of incision wound. **(B)** Rats thermal burn wounds model. The process of healing was followed and photographed at intervals of the 1st, 7th, 14th, and 28th day for the negative control group (Con), the treatment group (BO), and the positive group burn moisturizing scald (BMS), with 3 times/day for 28 days (n = 12 each group). Representative pictures revealing that BO pretreatment accelerate the healing process of thermal burn wounds. **(C)** The percentage of thermal wound contraction. BO pretreatment enhanced wound contraction observed in the rat thermal burn wounds model. The data were expressed as the mean ± SEM (n = 12 each group), compared with the control group, *p < 0.05, **p < 0.01.

The healing process of the incision wound was observed on the 1st, 5th, 10th, and 16th day after wounding, which is demonstrated in Figure 4A. The observation revealed that incision wounds treated with BO healed considerably faster in comparison with that of the Con on the 16th day. The wounds treated with BO initiated to form scar and shed the eschar gradually in contrast with the Con group. On the 16th day after the incision wound, the wounds of the BO group almost completely disappeared, whereas the scar in the Con group was still predominant. Consistent with the scar change, hair grew vigorously in the wound area in the BO-treated mice, which were entirely covered by the new epidermis.

The impact of BO on the healing process was also observed using the rat burn wound model, in which the percentage contraction ability on the 1st, 7th, 14th, and 28th day after wounding was recorded, as shown in Figure 4B,C. The result showed a significant (*p* < 0.01) increase in wound closure in the BO-treated group compared with the Con group on the 14th dayafter wound. On the last day, compared with the Con group, the scar of the BO group had entirely cured, and new fur had been growing on the scar, with the percentage of wound healing at 90.27 ± 2.06, which was significantly higher than that of the Con group (71.92 ± 5.92, *p* < 0.01). These results suggested that BO could promote wound healing.

### 3.6 Effects of BO on Skin Tissue Development

**FIGURE 5:**
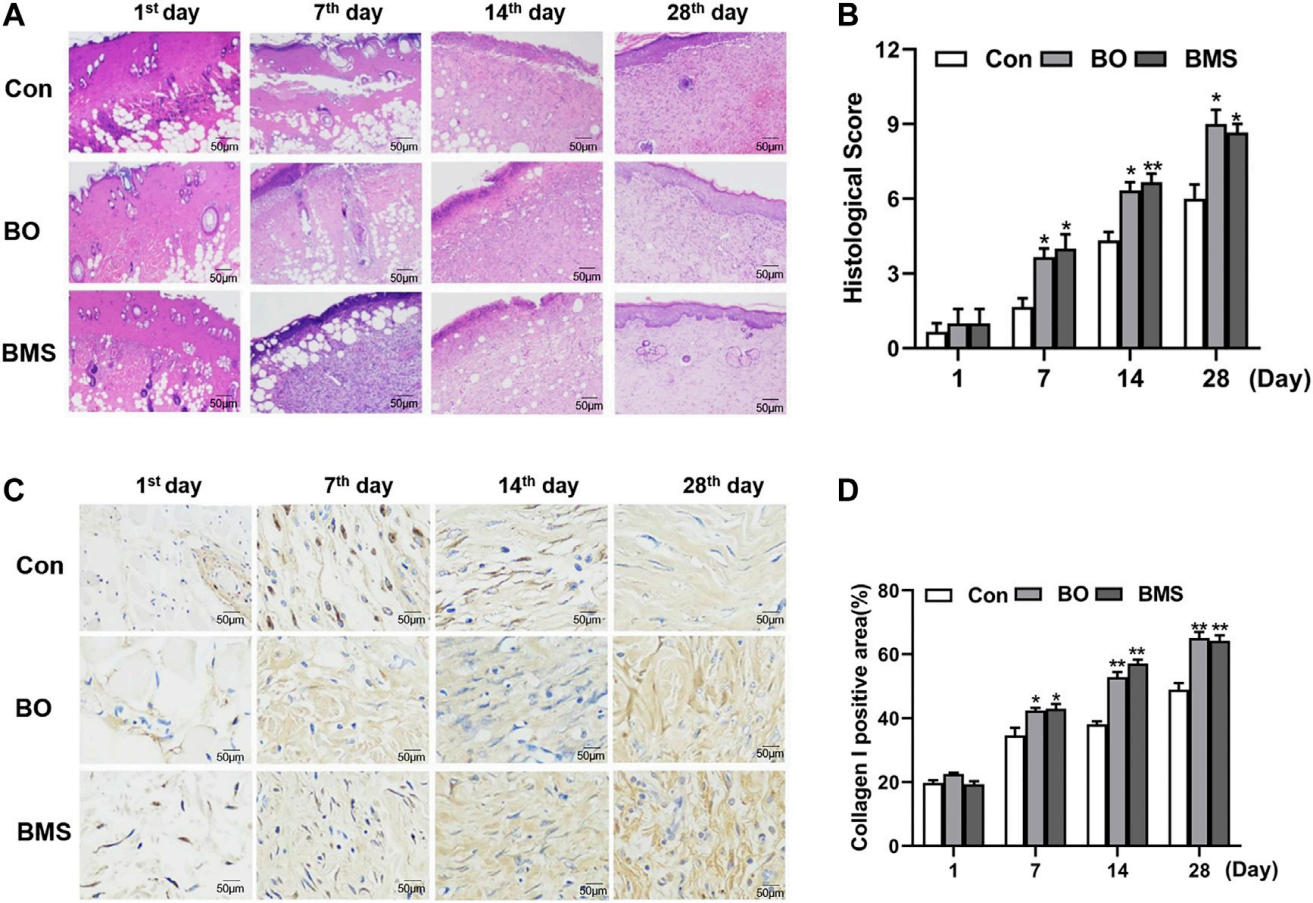
Histopathological evaluation and the type I collagen expression before and after Con, BO, and BMS treatment in the rat thermal burn wounds model. **(A)** Representative H&E imaging of scald wound in rats. Specimens were taken from the 1st, 7th, 14th, and 28th day after modeling. The negative control group (Con), the treatment group (BO), and the positive group burn moisturizing scald (BMS) (n = 12 each group). **(B)** The results of the histological score. Comparing with the Con group, the BO group’s wound was significantly healing on the 28th day (*p < 0.05), BMS group’s wound was prominently healing on the 14th day, and the data were expressed as the mean ± SEM, (*p < 0.05, **p < 0.01, respectively, n = 12 each group). **(C)** Increased type I collagen expression in the rat scald wound after BO treatment. Specimens were taken from the 1st, 7th, 14^th^, and 28th day after modeling. The negative control group (Con), the treatment group (BO), and the positive group burn moisturizing scald (BMS) (n = 12 each group). **(D)** The results of collagen Ι expression semiquantitation, compared with the control group. BO could upregulate the collagen Ι of wound area markedly from the 7th day; the data were expressed as the mean ± SEM (n = 12 each group); *p < 0.05, **p < 0.01, respectively.

The representative H&E images of the burn wound skin (100 × magnificent) are shown in Figure 5A, and the histopathological assessment is given in Figure 5B. The epidermis was fragmented, blood vessels and subcutaneous tissues under the dermis were severely damaged upon the burn wound creation. The BO-treated group healed faster than that of the Con group, with reduced inflammatory cell infiltration, increased fibroblast and blood vessels, and accelerated dermal and epidermal formation, at different time points. On the 28th day, the group treated with BO demonstrated an almost completely healed skin structure with normal epithelization, few inflammatory cell infiltrations, and the junction between the epidermis and dermis layers was tight and seamless when compared with the Con group. Moreover, the epidermis of rats treated with BO was much more uniform in thickness than that of the BMS group. The histopathological evaluation is shown in Figure 5B, which supported that the BO group was significantly improved than the Con group on the 7th, 14th, and 28th day (*p* < 0.05, respectively).

### 3.7 Effects of BO on Increasing Type I Collagen

The microscopic images of IHC stained burn wound skin (100× magnificent) and the percentage of collagen I changes are presented in Figure 5C,D, respectively. The results showed a significant increase (*p* < 0.05) in the percentage of collagen Ⅰ in the BO group when compared with the Con group. In the total study period of 28 days, the irregular collagen I areas of the BO group were increased and arranged tightly and evenly. In contrast, the group treated with canola oil was slowly increased. Similarly, the maximum positive area of collagen I observed was on the 28th day in the BO group (*p* < 0.01). The results indicated that BO played an important role in promoting an increase in cellular type I collagen.

### 3.8 Effects of BO on Regulating the Cytokine Production

**FIGURE 6:**
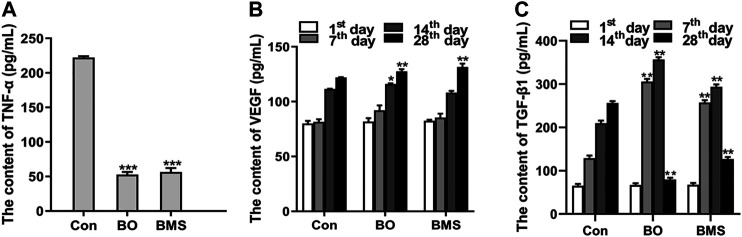
The serum cytokine level before and after Con, BO, and BMS treatment. **(A)** Downregulated TNF-α level after BO treatment. The data are expressed as the mean ± SEM (n = 12 each group), compared with the Con group, ***p < 0.001. **(B)** The content of VEGF, which was tested on the 1st, 7th, 14^th^, and 28th day. The data were expressed as the mean ± SEM (n = 12 each group), compared with the control group; *p < 0.05, **p < 0.01, respectively. **(C)** The content of TGF-β1, which was tested at the 1st, 7th, 14^th^, and 28th day. The data were expressed as the mean ± SEM (n = 12 each group), compared with the control group, **p < 0.01.

The TNF-α, VEGF, and TGF-β1 levels were detected by using the ELISA method, as shown in Figure 6. The levels of TNF-α were significantly lower after BO treatment than in the Con group (*p* < 0.001), indicating a potential anti-inflammatory effect of BO (Figure 6A).

The VEGF levels of the BO group were significantly increased on the 14th day compared with the Con group (*p* < 0.05). And the maximum significant differences (*p* < 0.01) of the content of VEGF between the BO-treated group and the Con group were obtained on the 28th day (Figure 6B). These results showed that BO could increase the content of VEGF in serum after scalding.

As presented in Figure 6C, in the first two weeks after wounding, the content of TGF-β1 of the BO group was significantly increased, compared with the content of the Con group. However, compared with the Con group on the 28th day, the content of the BO-treated group was significantly decreased. These results showed that BO could regulate the content of TGF-β1 in blood with time after burning.

### 3.9 Effects of BO on Activating the PI3K-AKT-mTOR Pathway

**FIGURE 7:**
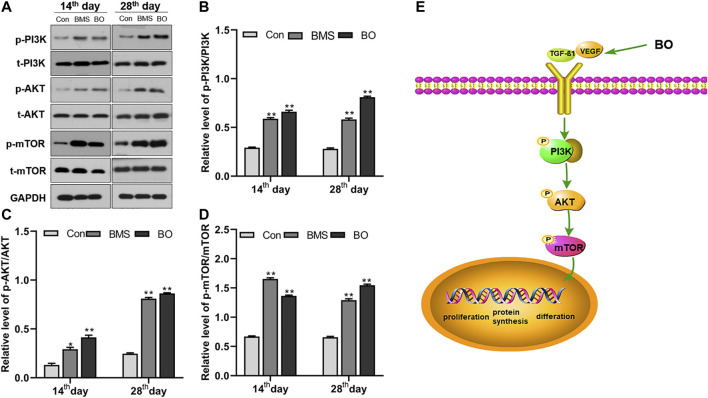
The ;xpression of the PI3K/AKT/mTOR signaling pathway proteins. **(A)** Representative Western blot bands of relevant proteins. Protein levels of **(B)** p-PI3K/t-PI3K, **(C)** p-AKT/t-AKT, and **(D)** p-mTOR/t-mTOR. Data were presented as the mean ± SEM of three independent experiments. Compared with the control group; *p < 0.05, **p < 0.01, respectively. **(E)** The illustrated picture of the BO-activated signal pathway within this study.

As is evidenced in Figure 7A, the application of BO resulted in increased phosphorylation of PI3K, AKT, and mTOR. Densitometry of bands showed that BO did not alter the expression of the unphosphorylated protein of PI3K, AKT, and mTOR. However, it significantly upregulated its phosphorylation compared with the Con group in the last two weeks after wounding (*p* < 0.01) (Figure 7B–D). These results suggested that BO could upregulate the expression of the PI3K/AKT/mTOR pathway.

## 4 Discussion

Burn and scald cause local damage to the body and systemic inflammatory response mediated by neuroendocrine (Gokakin et al., 2013), cytokine, and inflammatory mediators. These biomediators resulted in the delay of the healing of local burn and scald wounds and trigger of various, severe complications, such as pain, making treatment difficult (Naseri et al., 2018). The healing of burn and scald wounds involves three complex processes: inflammation, tissue hyperplasia, and regeneration. These three processes are not independent but overlap and develop in a particular order (Govindaraju et al., 2019). Exogenous damage will stimulate the body to secrete various growth factors, such as VEGF and TGF-β1, which can promote cell migration, proliferation, and differentiation, to help wound constriction and recovery. The damaged wound and these biomediators can also activate signaling pathways associated with wound repair, such as the PI3K/AKT/mTOR pathway (Cho et al., 2019; Zhang et al., 2020).

The inflammatory response is the first step after tissue damage and is essential for skin repair (Glim et al., 2015). A prolonged inflammatory response will be harmful to the body and lead to unexpected complications. Therefore, it is crucial to suppress inflammation and pain during tissue recovery. The clinical signs of inflammation are redness, swelling, heat, and pain (Yu et al., 2014). Swelling is a pathological manifestation of inflammation, and it is generally believed that eliminating swelling can achieve anti-inflammatory effects (Srivastava et al., 2018; Chen et al., 2019). To investigate the anti-inflammatory and analgesic effects of BO, we used a variety of classical animal models of inflammation, including mouse auricular swelling, rat toe swelling, and mouse hot plate test. These results showed that BO could inhibit auricular swelling in mice and toe swelling in rats (*p* < 0.05, *p* < 0.01, respectively), indicating its strong anti-inflammatory activity. In addition, the analgesic results showed that BO could relieve pain, and the pain threshold of BO-treated mice was significantly higher than that of control mice (*p* < 0.01), supporting the analgesic effect of BO.

When the wound presents with early inflammation, the body's defense system is activated, with the formation of a protective barrier and several growth factors production (Silvestre et al., 2018). TNF-α and VEGF are two of those cytokines and are critical in the wound healing process. TNF-α, one of the most vital proinflammatory cytokines, can induce vasodilatation, edema, and leukocyte adhesion to the epithelium. It can also regulate blood coagulation and contribute to oxidative stress in inflammation (Zelova and Hosek., 2013). Studies have shown that medications could decrease the release of inflammatory cytokines, such as TNF-α, reduce the inflammatory response, and promote wound healing (Weimann et al., 2018). In the present study, we found that BO could inhibit TNF-α release, which might be one potential anti-inflammatory mechanism of BO.

The secretion of VEGF in the healing process also exerts a significant effect, as VEGF can promote vascular endothelial cell proliferation and survival, accelerating the formation of new blood vessels (Peng et al., 2016; Wagner et al., 2016; Yan et al., 2017; Yen et al., 2018). The blood vessels are the channels of nutrient delivery in the body (Johnson and Wilgus, 2014), and it will ensure that the tissues and organs are provided with sufficient requirements for new skin formation (Sorg et al., 2018). Our results indicated that when the skin was scalded by high temperature, BO could upregulate the release of VEGF to facilitate wound healing. Also, collagen is necessary for repairing wounds. Studies had illustrated that TGF-β1 could regulate type Ι collagen gene expression and translation (Guo et al., 2018). However, the continuous increase of collagen Ι in the tissue will cause pathological scars to develop. Our results showed that the TGF-β1 content in the BO-treated group tended to increase early but decreased later. Based on these results, we speculated that with BO treatment, TGF-β1 stimulated the release of type I collagen at the early course to promote skin repair. At the later stage, to avoid collagen over-proliferation and scar formation, TGF-β1 expression is downregulated to inhibit the increase of collagen Ι.

The PI3K/AKT/mTOR signaling pathway is found in various cells and participates in cell growth, proliferation, apoptosis, and differentiation (Polivka and Janku., 2014; Peng et al., 2016; Dong et al., 2020). PI3K can be activated by growth factors, such as VEGF and TGF-β1 (Cui et al., 2017; Kim et al., 2018a; Zhang et al., 2019c). The activated state of growth factor receptors quickly phosphorylates phosphatidylinositol (4,5) P2 (PIP2) to obtain phosphatidylinositol (3,4,5) P3 (PI3P) (Zhang et al., 2019a). Protein kinase B (AKT) is one of the proteins downstream of PI3K; it is recruited by PI3P and phosphorylated by 3-phosphoinositide-dependent kinases (PDK-1) (Yudushkin, 2019). Then, p-AKT can regulate the protein level of phosphorylated mTOR to promote cellular growth and reproduction (Xu et al., 2018). Fibroblasts are a significant factor involved in wound healing. When tissue injury occurs, this signaling pathway is activated by increased VEGF and TGF-β1 levels, initiating fibroblasts migration to the wound site, proliferation, and differentiation into myofiibroblasts (Eming et al., 2014). These cells can help contract the wound border and contribute to producing collagen-like proteins, which are necessary for healing wounds and forming new skin tissue, accelerating the repair of burn wounds (Ohlund et al., 2014; Brenner et al., 2016; Xiao et al., 2017). Our Western blotting analysis showed that compared with the Con group, the BO-treated group had significantly increased activation of the PI3K/AKT/mTOR signaling pathway, with gradually upregulated phosphorylated proteins of this pathway. This finding suggested that the preliminary mechanism by which BO promoted burn repair might be related to the activation of the PI3K/AKT/mTOR signaling pathway.

In order to evaluate the quality and standard of preparations of BO, we detected and analyzed eight quality marker components in BO by HPLC, which contained aloe emodin, emodin, rhein, chrysophanol, ferulic acid, lobetyolin, asarinin, and borneol. The active compounds aloe emodin, emodin, rhein, and chrysophanol were reported to be quality markers of *Rheum palmatum* L. with anti-inflammatory activity and to promote wound healing, and their pathways of action may be involved in the proliferation and differentiation of skin epithelial cells through the regulation of PI3K/AKT pathway (Dong et al., 2016; Wu et al., 2017; Dong et al., 2019; Su et al., 2020). Ferulic acid is a quality marker of *Angelica sinensis* (Oliv.) Diels, which can promote wound healing (Wei et al., 2016). Lobetyolin is a quality marker of *Codonopsis pilosula* (Franch.) Nannf. Ferulic acid and lobetyolin can increase the content of VEGF in blood vessels and promote angiogenesis (Lin et al., 2010; Wang et al., 2020). Asarinin, a quality marker of *Asarum sieboldii* Miq., increases TGF-β content, reduces toe swelling, and regulates inflammation in arthritis model mice through the NF-κB pathway (Dai et al., 2019). And *Borneolum syntheticum*, whose chemical component is borneol, is a natural transdermal absorption enhancer that promotes BO absorption (Dai et al., 2018).

All of the above compounds have been reported to exhibit anti-inflammatory, analgesic, and skin healing promoting effects individually, but the combined effects of these compounds have not been investigated. In the present report, we investigated the role and mechanism of BO containing a mixture of active ingredients in anti-inflammatory and skin healing promotion. Our study showed that BO had a promising effect, which was most likely achieved by modulating the P13K/AKT transduction pathway.

## 5 Conclusion

In conclusion, this study demonstrated that BO is a pharmacologically active traditional Chinese medical formulation for promoting wound healing. It can accelerate wound healing through PI3K/AKT/mTOR signaling pathway and increase the key cytokines VEGF and TGF-β1 to further help tissue recovery. This study provides an insight into the in vivo effects of this preparation and its mechanism of action of BO in the treatment of burns and scalds, which will provide some guidance for the clinical application and development of BO.

## Data Availability

The original contributions presented in the study are included in the article/ further inquiries can be directed to the corresponding authors.

## Abbreviations

AKT, protein kinase B; AST, aspartate transaminase; BMS, burn moisturizing scald ointment; BO, burn ointment; Cr, creatinine; DAB, diaminobenzidine; ELISA, enzyme-linked immunosorbent assay; H&E, hematoxylin-eosin; GAPDH, glyceraldehyde phosphate dehydrogenase; mTOR, mammalian target of rapamycin; PAGE, SDS-polyacrylamide gel electrophoresis; PBS, phosphate buffered saline; PDK-1, 3-phosphoinositide-dependent kinases; PI3K, phosphatidylinositol 3-kinase; PIP3: phosphatidylinositol (3,4,5) P3; PVDF, polyvinylidene difluoride; SDS, sodium dodecyl sulfate; TCM, traditional Chinese medicine; TGF-β1, transforming growth factor-beta1; TNF-α, tumor necrosis factor-alpha; VEGF, vascular endothelial growth factor; PIP2, phosphorylates phosphatidylinositol (4, 5) P2.
